# Change in Conductive–Radiative Heat Transfer Mechanism Forced by Graphite Microfiller in Expanded Polystyrene Thermal Insulation—Experimental and Simulated Investigations

**DOI:** 10.3390/ma13112626

**Published:** 2020-06-09

**Authors:** Aurelia Blazejczyk, Cezariusz Jastrzebski, Michał Wierzbicki

**Affiliations:** 1 Department of Mechanics and Building Structures, Institute of Civil Engineering, Faculty of Civil and Environmental Engineering, Warsaw University of Life Sciences—SGGW, Nowoursynowska 159 ST., 02-776 Warsaw, Poland; 2 Faculty of Physics, Warsaw University of Technology, Koszykowa 75 ST., 00-662 Warsaw, Poland; cezariusz.jastrzebski@pw.edu.pl (C.J.); michal.wierzbicki@pw.edu.pl (M.W.)

**Keywords:** expanded polystyrene, graphite particles, thermal conductivity, thermal resistance, thickness effect, phonon-photon transport, Raman spectroscopy, thermal analysis

## Abstract

This article introduces an innovative approach to the investigation of the conductive–radiative heat transfer mechanism in expanded polystyrene (EPS) thermal insulation at negligible convection. Closed-cell EPS foam (bulk density 14–17 kg·m^−3^) in the form of panels (of thickness 0.02–0.18 m) was tested with 1–15 µm graphite microparticles (GMP) at two different industrial concentrations (up to 4.3% of the EPS mass). A heat flow meter (HFM) was found to be precise enough to observe all thermal effects under study: the dependence of the total thermal conductivity on thickness, density, and GMP content, as well as the thermal resistance relative gain. An alternative explanation of the total thermal conductivity “thickness effect” is proposed. The conductive–radiative components of the total thermal conductivity were separated, by comparing measured (with and without Al-foil) and simulated (i.e., calculated based on data reported in the literature) results. This helps to elucidate why a small addition of GMP (below 4.3%) forces such an evident drop in total thermal conductivity, down to 0.03 W·m^−1^·K^−1^. As proposed, a physical cause is related to the change in mechanism of the heat transfer by conduction and radiation. The main accomplishment is discovering that the change forced by GMP in the polymer matrix thermal conduction may dominate the radiation change. Hence, the matrix conduction component change is considered to be the major cause of the observed drop in total thermal conductivity of EPS insulation. At the microscopic level of the molecules or chains (e.g., in polymers), significant differences observed in the intensity of Raman spectra and in the glass transition temperature increase on differential scanning calorimetry(DSC) thermograms, when comparing EPS foam with and without GMP, complementarily support the above statement. An additional practical achievement is finding the maximum thickness at which one may reduce the “grey” EPS insulating layer, with respect to “dotted” EPS at a required level of thermal resistance. In the case of the thickest (0.30 m) panels for a passive building, above 18% of thickness reduction is found to be possible.

## 1. Introduction

Large-scale application of EPS foams with closed cells as thermal insulation in construction engineering requires the sustainable improvement of the thermophysical features of traditional building materials [1,2,3,4,5]. When introducing various technological changes in chemical composition, besides improving measurement and simulation techniques, it is important to understand the physical consequences caused by such changes. A better understanding may allow for the generation of knowledge, as well as let scientists improve the modelling of thermal processes. Moreover, it may allow engineers to develop novel materials and encourage industry stakeholders by more effectively optimizing the production costs of these materials, along with better thermal insulation performance.

A traditional solution for high quality thermal protection in buildings is by using high-thickness insulating layers made of conventional thermal insulation that, together with other more advanced options, comprises “the best building practice”. Advanced thermal insulation materials, such as super insulating materials (SIMs) (e.g., vacuum insulation materials (VIMs) and aerogels), phase change materials (PCMs), gas-filled materials (GFMs), and nanoinsulation materials (NIMs), promise to be the most beneficial for applications in the building sector [6,7,8]. In addition, modern systems, such as adaptive facades, dynamic facades, and active envelopes, provide architectural alternatives, which are a novel platform for energy efficiency, visual comfort, and daylight distribution, as well as branding and image. However, installing conventional thermal insulation of high thickness in external walls remains an attractive option, especially in harsh climate countries, due to the low market prices of materials and the costs of their installation [9,10,11,12]. When designing a building, one of the first steps to consider is reaching the required level of thermal protection, in terms of thermal transmittance *U*-value, which ranges from ca. 0.1 W·m^−2^·K^−1^ for high-energy standards up to 3 W·m^−2^·K^−1^ or more for low-energy standard [13]. The challenge is to obtain such values by selecting the most appropriate and cost-effective materials, which provides the thinnest possible insulation with the highest thermal effectiveness.

Conventional polymeric foams applicable in the building sector include both products of very low bulk density (below 20 kg·m^−3^), such as extruded polystyrene (XPS) or EPS (herein under study), and products of low bulk density (up to 45 kg·m^−3^), such as phenolic foam (PhF), polyurethane (PUR), and polyisocyanurate (PIR) foams, as well as XPS and EPS foams. A popular option is the industrial production of polymer composite, such as EPS foams that include graphite fillers [14,15,16], due to low cost, lightweight, and moderate dispersibility in polymer matrix [17,18], in particular, graphite microparticles (GMP) [19,20,21,22,23,24,25]. These are typically applied in the form of panels for the thermal insulation of buildings. Despite the problem of possible overheating due to solar radiation causing panel deformation [26,27], this choice offers designers relevant insulation thickness reduction. The thickness of “pure” EPS panels in the external walls of passive buildings (*U*-value = 0.1 W·m^−2^·K^−1^ for Central European climates) can reach up to *d* = 0.30 m. As it is of interest to reduce the thickness (at a given *U*-value), one may study the material’s thermal insulation performance [28,29,30]. Some optimization is possible by observing heat transfer via the apparent thermal conductivity *λ*′ and resistance *R*′ as a function of the EPS sample thickness *d*, with relation to the variable bulk density *ρ* and concentration of fillers, such as GMP.

Following measurements in accredited laboratories, EPS manufacturers have declared its thermal conductivity coefficient *λ*_D_ and thermal resistance *R*_D_ values [31,32]. However, the literature has barely discussed the so-called thickness effect, which has the apparent impact of the sample thickness (in its lower range) on the material’s thermophysical parameters [33,34]. On the other hand, the thickest samples have rarely been measured and reported [35]. The latter may be due to common technical difficulties when measuring insulation panels above 0.1 m in thickness, which is not suitable for most of the (too narrow) plate instruments (GHP and HFM). Besides the main purpose of thermal transport analysis, this work also tries to fill this gap, explaining these technical issues (i.e., experimental knowledge regarding thin and thick case measurements). Therefore, the extra purpose is demonstrating a methodical approach that may answer the following two questions:

- How can we overcome the experimental artefact of the thickness effect, as revealed by conventional polymeric foam panels?
- How can we find the true conductivity *λ* and resistance *R* values for the panels which are thicker than the gauge of plate instruments?

In each polymeric insulating material, one must initially assess the thickness effect relevance by testing thin slices of the foam panel. Herein, it is done, concerning EPS of very low bulk density, by using an experimental method in accordance with the recommendations of the European Committee for Standardization CEN, as described in Standards [30,32,35,36].

Besides considering technical issues and providing correct data for engineers, this paper aims to contribute to the general discussion about the thermal transport mechanisms in polymeric insulating materials and better understanding them. Example of such discussions describing mainly EPS insulation can be found in the recognized literature [37,38,39,40,41,42,43,44,45,46,47,48,49]. In particular, it has been reported that, when incorporating industrially higher concentrations of GMP into an EPS polymer matrix, the total thermal conductivity can be reduced by opacifying the insulation product for thermal radiation, which is absorbed by the carbon atoms in graphite or scattered by the larger graphite microparticles. Unfortunately, the quantitative analysis of the three major thermal components—radiation, air conduction, and polymer matrix conduction—over a wide thickness range of the insulating EPS layers with different GMP industrial concentrations is missing, in general.

Therefore, the main scientific purpose is analysing heat transfer through EPS foam of very low bulk density and separating it into its radiative and conductive thermal components. To the authors’ knowledge, this is the first successful approach of separating thermal components to examine EPS in relation to the identified spectral features of the Raman spectrum. As for convection of insulating gas, it is proved to be negligible in EPS, as well as also other polymeric foams with a closed or open-cell structure of diameter up to 0.004 m (4 mm) [1,27,50,51,52]. The closed cells in the expanded beads of “pure” EPS at various densities are of size ranging from 0.05 to 0.40 mm [27,38,53]. The scientific aim of the present paper was achieved by comparing experimental and literature data [42]. As a result, we observe the effect of change in conductive–radiative heat transfer mechanism triggered by the GMP at two different industrial concentrations. Its apparent impact on each particular thermal component is then analysed and discussed.

## 2. Materials and Methods

This section briefly outlines characteristics of the tested EPS products and describes the experimental methods used to measure their physical parameters, including the accuracy/uncertainty estimation in the error analysis, as well as comments on the experimental limitations.

### 2.1. Tested Products

**Figure 1:**
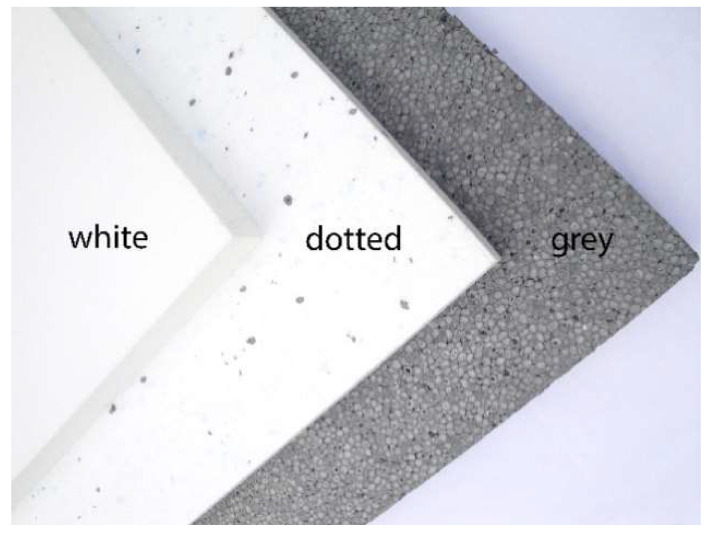
The tested products: A—“white” expanded polystyrene (EPS) (pure), B—“dotted” EPS, and C—“grey” EPS.

The tested EPS products were manufactured by Polish enterprises, in the form of panels, for thermal insulation of buildings. The three types of commercial EPS products investigated were (Figure 1):

- “white” EPS A—pure;
- “dotted” EPS B—with a low concentration of GMP (only within the expanded beads of the black dotted isles, scattered randomly in the entire panel); and
- “grey” EPS C—with a high concentration of GMP (evenly distributed throughout the polymer matrix and in the entire panel).

It is known, from the literature [45], that the size of industrially applied GMP ranges from 1 to 15 µm (diameter distribution range).

The GMP mass concentration in EPS C can be estimated, from measured density values, as equal to the relative density change (*ρ*_C_ − *ρ*_B_)/*ρ*_C_. By neglecting the GMP concentration in the EPS B product, a rough prediction of the graphite content in EPS C is up to 7.3%, as ((14.6 + 0.5) − (14.2 − 0.2))/(14.6 + 0.5) = 0.073 (see Section 3.1 Table 2; also confront with Section 3.6).

Each of the tested EPS panels was made by cutting a single massive block (material batch) by a hot wire to the desired thickness on a manufacturing line. The range (from 0.02 to 0.18 m) and the particular thicknesses of the individual panels can be read in Section 3.1 from Figure 3. Then, the panels were subjected to systematic control of their thermal insulation performance. Unlike B and C, the thermal parameters of A were unstable during the first 20 months of seasoning. During the entire experiment, the panels were conditioned under stable laboratory conditions: air temperature (20 ± 2 °C) and relative air humidity (50 ± 10% RH).

### 2.2. Experimental Limitations for Thermal Measurement

The first experimental limitation arises from the fact that testing the thermal insulation performance of any homogeneously porous material can be conducted using any plate instrument, but only if the maximum nominal size of any diversity in its structure (i.e., grains or pores—whatever is larger) is smaller than one-tenth of the sample thickness [28,29,36]. Slightly varying with bulk density [27,39,40,44], the order of magnitude of the “pure” EPS grains (expanded beads filled with closed cells) size may reach 10^−3^ m; then, the minimum thickness of the EPS sample should be at least 10 times greater, which sets the minimum thickness no less than 10^−2^ m.

The second limitation arises from determining the thermal parameters of EPS of very low or low bulk density. Regarding the experimental method, this is related to the so-called thickness limit estimation [35], which is further explained in Section 2.4.1 and Supplementary Material part 1.

The third limitation is caused by the so-called permissible sample thickness range (from minimum *d*_min_ to maximum *d*_max_), depending on the specific plate instrument chamber dimensions and the measurement section area. This range is suggested by the Standards [28,29,30,35].

### 2.3. Bulk Density Measurement Method

The bulk density *ρ* was determined experimentally for the tested EPS products by measuring (for each panel thickness *d*):

- the panel mass, with an accuracy of ±10^−5^ kg,
- the panel dimensions (length *x* and width *y*), with an accuracy of ±5 × 10^−4^ m; and thickness *d*, with an accuracy of ±10^−6^ m,

and calculating:

- the panel volume (as a regular rectangular prism), with accuracy no worse than ±10^−4^ m^3^ and
- the panel density (as the mass to volume ratio) and the bulk density of the material (as an average over the thickness range), with accuracy no less than ±2 × 10^−1^ kg·m^−3^.

A standard precise laboratory balance (Radwag PS 2100.R1) was used for mass measurement. The length and width were found using a professional EC Class 1 measuring tape. The thickness value was given by an HFM measuring system. The environment was controlled, in terms of air temperature (20 ± 2 °C) and relative air humidity (50 ± 10% RH).

Details of the expanded uncertainty calculations regarding the bulk density measurements are explained in Supplementary Material part 2. The averaged expanded uncertainty <*U*(*ρ*)> values of the bulk density *ρ*(*d*) measurement were calculated, from all experimental uncertainties *U*(*ρ*) within the whole thickness range under study, individually for each type of EPS product. The results are given in Section 3.1.

### 2.4. Thermal Measurement Method

This section describes a practical approach for the measurement of the thin and thick EPS panels, which takes into consideration the limitations mentioned in Section 2.2, as well as an experimental setup that allows us to obtain correct thermal measurements as output.

#### 2.4.1. The Thickness Effect and Thickness Limit of (Non-)Linearity

The thickness effect refers to the apparent sublinear growth of the apparent thermal conductivity *λ*′(*d*), up to a certain level achieved at the critical thickness limit *d*_L_, above which the function takes a seemingly constant value (yet still below the upper bound—herein assigned to the thermal transmittance *λ*′_t_). Above *d*_L_, the value of *λ*′(*d*) approaches the true material conductivity *λ*(*d*), so one may assign the values of *λ*(*d*) to the values of *λ*′(*d*). The data seem to oscillate around <*λ*> (the material-representing averaged value).

The thickness limit, *d*_L_, results from several factors, such as the material property, sample features, the experimental set-up, and so on. The limit could be assigned to the minimal thickness above which the thermal transmittance *λ*′_t_ can be determined from the thickness-independent ratio Δ*d*/Δ*R*′ [28,29,33,36]. As *λ*′_t_ corresponds to the inverse gradient of the oblique asymptote of *R*′(*d*), it can be determined by a linear fit to all data above *d*_L_.

When *d* > *d*_L_, the so-called transfer factor *ℑ* (see Supplementary Material part 1) practically does not depend on the thickness (within experimental inaccuracy tolerance). In this region, *ℑ* does not differ from *λ*′_t_ by more than 2% [28,29,36]. In other words, in the case of sufficiently large thermal insulation thickness, the asymptotic values of *λ*′(*d*) and *ℑ*(*d*) are equal to the value of *λ*′_t_. Thus, the level of *λ*′_t_ is the limiting horizontal asymptote achieved by both functions.

As shown in Supplementary Material part 1, for the EPS A, B, and C products, the respective thickness limits *d*_LA_, *d*_LB_, and *d*_LC_ can be obtained by comparing the thickness-dependent 1 − *L*(*d*) values (which are linearity measures of the *λ*′ or *R*′ curves at a given *d*) with the experimental tolerance for nonlinearity of the measured data at the optimum level of 0.02. In general, the lower the level of 1 − *L*, the larger the value of *d*_L_; thus, as 1 − *L* approaches 0.00, the thickness limit tends to infinity, which means that none of the EPS products could comply with the requirements and determination of their thermal parameters would be impossible. If a less rigorous condition, such as 1 − *L* ≤ 0.03, is applied, then the resultant *d*_L_ would obviously be inside of the curved region. If a more rigorous condition, such as 1 − *L* ≤ 0.01, is applied, then the thicknesses limits would be unreasonably far from the curved region. Thus, by applying the optimal experimental condition of 1 − *L* ≤ 0.02, one can finally obtain the reasonable thickness limits (see Section 3.2).

The thickness effect has an impact not only on the measurement of thin panels but also on the thickest panels, exceeding the maximum distance *d*_max_ between “hot” and “cold” plates in the HFM instrument. In the latter case, the determination of the material thermophysical parameters *R* and *λ* could be achieved by the proposed procedure, which includes cutting thinner panels from the thick product block (to a thickness no smaller than *d*_L_), measuring the *R*′(*d*) value, correcting data and extrapolating the obtained values of *R*(*d*) up to the considered product thickness and, finally, conversion from resistance *R* to conductivity *λ* of the product. Nevertheless, before cutting the panels, one has to take into consideration the thickness effect, evaluating its relevance by calculation of *d*_L_. Once the thickness limit is known, the total thermal resistance of the product block can be calculated (even without *R*′(*d*) correction), according to the practical formula (1):

(1) $$R_{\mathrm{product}}^{\mathrm{EPS}}=\frac{\sum_{i=1}^{n}\frac{R_{i}}{d_{i}}}{n}\cdot d_{\mathrm{product}}^{\mathrm{EPS}}$$

where i = 1, 2, …, n and n is the number of panels cut from the block of product under consideration, where the condition *d* ≥ *d*_L_ is satisfied for each panel.

#### 2.4.2. HFM Setup

Thermal tests in steady state conditions were carried out by using the HFM FOX 600 plate instrument, made in the USA by the LaserComp Company.

As mentioned in Section 2.2, the Standards recommend limiting thermal measurements to a permissible sample thickness range. These limits depend on the geometry of the experimental setup. The HFM chamber dimensions were 0.600 × 0.600 m^2^, the dimensions of its measuring section area (located at the heating/cooling black plate centre) were 0.300 × 0.300 m^2^ (also assigned to the size of the sample area), and the dimensions of the tested panels were 0.600 × 0.500 m^2^. In this case, the permissible thickness should range from *d*_min_ = 0.030 m to *d*_max_ = 0.150 m [30]. Yet, as has been demonstrated, based on uncertainty analysis, the thermophysical parameters obtained for samples between 0.020 and 0.180 m were reliable. In general, the HFM allows for mounting and precisely measuring samples of thickness from 0.005 m (see Section 3.4) up to 0.200 m (max gauge space).

The HFM was calibrated using the certified Standard Reference Material (SRM–IRMM–440), as recommended by the Institute for Reference Materials and Measurements (IRMM) in Geel. The SRM characteristics are shown in Table 1, for comparison of the measurements completed by IRMM in Geel with the author’s results. The tiny value of the correction parameter *𝒞*, resulted even less than *U*(*λ*^C^_SRM_), reflecting proper experimental setup and testing conditions.

In order to estimate the thermal radiation component (through the insulation), the surface emissivity values individually for the HFM black plate and 10 µm Al-foil were measured earlier at *T*_m_ = 10 °C (no sample inside the HFM chamber), according to the Standard [30]. The latter measurement was carried out by placing two symmetrical Al-foil layers attached to the HFM bottom and the top black plate. The resulting values were as follows: 0.873 for the HFM black plate, 0.042 for the rough Al-foil side, and 0.032 for the polished Al-foil side.

#### 2.4.3. Conductivity-Resistance Measurement Method

##### Standard Method

Measurements of thermal insulation performance of the tested EPS products were carried out in compliance with the recommendations of the Standards [28,29,32,33,35] and the acquired data processing (for the conductivity and resistance correction) was completed according to the relations and procedure described in Supplementary Material part 1.

**Figure 2:**
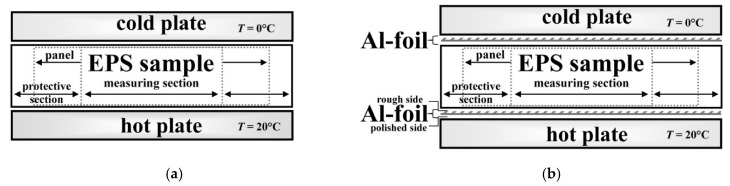
Schematic design of the measurement system in: (**a**) standard and (**b**) nonstandard method.

The environment was controlled, in terms of air temperature (20 ± 2 °C) and relative air humidity (50 ± 10% RH). The HFM was set to the average test temperature *T*_m_ = 10 °C. The temperature difference applied to the sample was Δ*T* = 20 °C. The heating (bottom) black plate temperature was set as 20 °C and the cooling (top) black plate temperature was set as 0 °C, such that the heat flow was directed upwards. A schematic design of the measurement system in standard method is shown in Figure 2a. For each EPS panel tested (see Section 2.1), the following parameters were measured (i.e., the HFM output):

- the sample thickness *d*, with accuracy Δ*d* of ±10^−6^ m;
- the temperature difference Δ*T* between upper and lower sample surfaces, with accuracy Δ(Δ*T*) of ±0.1 °C;
- the heat flux density *q* through the sample, with accuracy Δ*q* of ±10^−1^ W·m^−2^;
- the apparent thermal resistance *R*′, with accuracy Δ*R*′ of ±10^−4^ m^2^·K·W^−1^; and
- the apparent thermal conductivity coefficient *λ*′, with accuracy Δ*λ*′ of ±10^−5^ W·m^−1^·K^−1^.

Details of the expanded uncertainty calculations regarding the thermal conductivity measurements are explained in Supplementary Material part 2. The average expanded uncertainty <*U*(*λ*)> values of *λ*(*d*) were calculated from all experimental uncertainties *U*(*λ*), within the whole thickness range under study, individually for each type of EPS product. The results are given in Section 3.3.

##### Nonstandard Method

Furthermore, during nonstandard HFM measurement, i.e., the sample placed between two Al-foil layers (applied at the bottom and on the top of the insulation) and HFM plates, as shown in Figure 2b, one may try to assume that the expected level of the total thermal conductivity may be considered as the system response, in which radiation (primary—external, emitted by the HFM “hot” plate and secondary—internal, radiation generated across the insulation) is sufficiently blocked (cut off) by reflection from the bottom and upper Al-foil, respectively. However, from primary continuous radiative–conductive heat flux, only phonons can pass through the Al-foil (either the “hot” or the “cold” one). One must notice that the Al-foil emissivity value (see Section 2.4.2) is no more than 0.04 and that above 96% of the photon flux, either “primary” or “secondary”, can be reflected back by the upper Al-foil (in the extreme scenario). Hence, in order to estimate the total thermal conductivity with simulated Al-foil effect, only for the tested EPS B and C products, the difference between the heat flux without Al-foil and the heat flux with Al-foil may correspond to the thermal radiation component, which allows us to calculate its contribution to the total thermal flux individually for each product. This topic is presented further in Section 4.1.2 and Supplementary Material part 3, also explaining the impact of GMP on total thermal conductivity.

### 2.5. Micro-Raman Measurement

The micro-Raman Spectroscopy (µ-RS) measurements were carried out by using Renishaw’s inVia Reflex Spectrometer. The µ-RS tests were performed to investigate comprehensively microstructural changes (e.g., at the molecular level) of the insulation samples. The Raman spectra were collected at room temperature and normal conditions, in backscattering geometry with the 633 nm line of a He–Ne-ion laser and with the 514 nm line of an Ar-ion laser as excitation wavelengths. The results are given in Section 3.5.

### 2.6. Thermal Analysis Measurement

Thermogravimetric analysis (TGA) and differential scanning calorimetry (DSC) measurements were carried out by using TA Instruments equipment i.e., SDT Q600 and Q2000, respectively.

The conventional DSC tests were performed to measure the amorphous glass transition (*T*_g_) or the crystalline melting (*T*_cm_) temperatures of the insulation samples. The DSC thermograms were collected at the temperature range of −100 to 350 °C at a heating rate of 10 °C·min^−1^ and a nitrogen flow rate of 50 mL·min^−1^. In order to reduce the impact of pressure increase on the measurement results, the samples were put into the nonhermetic aluminium calorimetric containers; appropriate empty aluminium containers were used as reference. The temperature scale was calibrated with the melting point of indium. The estimated error in the determination of *T*_g_ or *T*_cm_ was ±2 °C.

The TGA tests were performed to measure temperature dependence of the mass loss of the insulation samples. The TGA thermograms were collected at the temperature range of 25–550 °C, at a heating rate of 10 °C·min^−1^, a nitrogen flow rate of 100 mL·min^−1^, and in the hermetic aluminium containers. The results are given in Section 3.6.

## 3. Experimental Results

### 3.1. Bulk Density and Homogeneity Assessment

**Figure 3:**
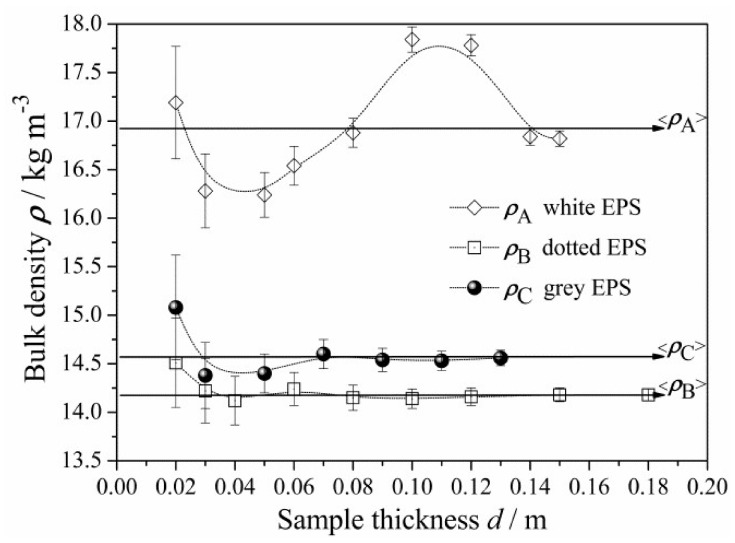
The measured bulk density *ρ* versus the panel thickness d for the EPS A, B, and C products. The error bars correspond to the expanded uncertainties, *U*(*ρ*). The horizontal arrows indicate the levels of the average density <*ρ*> values (see Table 2).

Each measured bulk density *ρ*(*d*) value, representing an EPS panel of a given *d*, was found as the average of several single measurements and plotted, with a vertical error bar corresponding to its expanded uncertainty, *U*(*ρ*) and a horizontal error bar covered by the symbol used in Figure 3.

Next, the averaged bulk density <*ρ*> was calculated, from all experimental *ρ*(*d*) values, individually for each tested EPS product. The resulting <*ρ*_A_>, <*ρ*_B_>, and <*ρ*_C_> values are reported in Table 2, including average expanded uncertainties <*U*(*ρ*)>, and presented in Figure 3.

The tested EPS A density was slightly higher, while the EPS B and C products revealed comparable density values.

Homogeneity assessment for each tested EPS product was performed based on *ρ*(*d*) measurements, throughout the entire thickness range. As seen from Figure 3 and Table 2, the *ρ*_A_(*d*) points for the EPS A product reveal the widest spread of bulk density, extremely fluctuating around the average value <*ρ*_A_>; furthermore, its corresponding standard deviation was the largest. This indicates the relatively poor homogeneity (possibly due to differences in density between the pre-expanded beads and expanded beads, which were mixed for recycling purposes during the final block foaming process [4,43,54,55]). On the contrary, EPS B was the most homogenous product (no recycling, in this case).

### 3.2. Thickness Limits Results

All basic calculations of the thermophysical parameters, including the heat transfer factor *ℑ*, the thickness effect function *L*(*d*), and the thickness limit *d*_L_, are elaborated and shown in Supplementary Material part 1.

In order to determine the thermal insulation performance of each tested EPS product, first and foremost, one has to check whether the thickness effect is relevant. This analysis, performed separately for each EPS type, was based on testing the values of the function *L*(*d*) over the investigated thickness range. Wherever 1 − *L*(*d*) > 0.02 (see Section 2.4.1), at lower thicknesses, the effect was qualified as relevant (thermal conductivity and resistance were apparently nonlinear functions of thickness); wherever 1 − *L*(*d*) ≤ 0.02, at higher thicknesses, the thickness effect was qualified as irrelevant (thermal conductivity and resistance were nearly linear functions of thickness).

**Figure 4:**
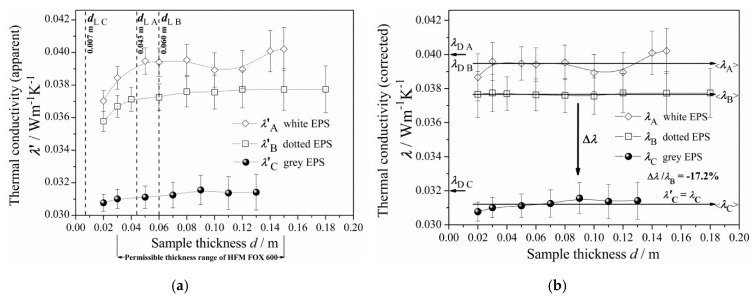
(**a**) The apparent thermal conductivity coefficient λ′ versus the panel thickness d for the EPS A, B, and C products, as measured at *T*_m_ = 10 °C. The error bars correspond to the expanded uncertainties *U*(*λ*′). The vertical dashed lines, labelled by d_LA_, d_LB_, and d_LC_, show the corresponding thickness limits; (**b**) The corrected thermal conductivity coefficient *λ* at *T*_m_ = 10 °C versus the panel thickness d for the EPS A, B, and C. The error bars correspond to the expanded uncertainties *U*(*λ*). The horizontal right arrows indicate the average conductivity <*λ*> values from Table 3. The left arrow on the level 0.04 indicates *λ*_DA_ and *λ*_DB_ (as declared for the A and B products), while *λ*_DC_ appears at the 0.032 level. The vertical down arrow Δ*λ* shows the effect of graphite microparticles (GMP) on thermal conduction. Referring EPS C to EPS B, the relative drop of conductivity achieved was 17.2%.

In the case of EPS A and B, the thickness effect appeared to be relevant up to the estimated limits (*d*_LA_ and *d*_LB_) shown in Section 3.3 (Table 3 and Figure 4a). As can be seen, in the case of EPS C, the thickness effect appeared to be irrelevant (negligibly small), as the estimated limit *d*_LC_ appeared to be lower than the permissible minimum *d*_min_ of the HFM.

### 3.3. The Thermal Conductivity Results

The declared thermal conductivity coefficient, *λ*_D_, taken from technical data sheet (TDS) of each tested EPS product, was compared with the average corrected thermal conductivity <*λ*> (see Table 3 and Figure 4b). The <*λ*_A_>, <*λ*_B_>, and <*λ*_C_> values, together with their uncertainties <*U*(*λ*)>, represent the tested EPS products of any thickness, which were all calculated from *λ*(*d*), respectively.

Each collected thermal conductivity value *λ*′(*d*), representing an EPS panel of given *d*, was found as the average of several single measurements and was plotted, with vertical error bar corresponding to its expanded uncertainty, *U*(*λ*′) and horizontal error bar covered by the symbol used in Figure 4a.

Next, due to relevance of the thickness effect revealed by the EPS A and B products, partial corrections of their apparent thermal conductivities *λ*′(*d*) were performed, according to the Standards [32,33,35] (see Supplementary Material part 1). Figure 4b shows the corrected thermal conductivity *λ*(*d*) for EPS A, B, and C. The error bars correspond to the *U*(*λ*) values. For EPS C, the data was not modified, such that *λ*′(*d*) = *λ*(*d*). As seen in Figure 4b, in each case of EPS A, B, and C, the *λ*(*d*) values oscillate around <*λ*> and below the *λ*_D_ level within the whole thickness range. Each obtained result satisfies the standard inequality:

(2) $$\lambda_{D}\geq 0.44\sigma +\langle \lambda \rangle$$

which is used to qualify the material as complying with requirements of the Standards [31,32].

In order to show the effect of the GMP concentration on the total thermal conductivity, the absolute change in thermal conductivity coefficient (comparing the EPS C and B products), was defined as difference:

(3) $$\Delta \lambda =\lambda_{C}-\lambda_{B}$$

It is shown as a vertical down arrow in Figure 4b. Also, the relative change in thermal conductivity coefficient was defined as:

(4) $$\frac{\Delta \lambda }{\lambda_{B}}=\frac{\lambda_{C}-\lambda_{B}}{\lambda_{B}}$$

Both Δ*λ* = −6.5 × 10^−3^ W·m^−1^·K^−1^ and Δ*λ/λ*_B_ = −0.172 have constant negative values within the whole range of panel thickness. Thus, the total thermal conductivity of the “grey” EPS C was about 17.2% smaller than that of the “dotted” EPS B. This result was very close to the literature data, comparing “grey” and “pure” EPS [37,43] of bulk density ca. 14–17 kg·m^−3^. Cautiously comparing the “grey” EPS C and “pure” EPS from [37] of comparable bulk density ca. 14 kg·m^−3^ results in −25% change. Thus, the total thermal conductivity of the “dotted” EPS B was about 7.8% smaller than of the “pure” EPS from [37].

The results are discussed further in Section 4.1.

### 3.4. The Thermal Resistance Results

The declared thermal resistance, *R*_D_, as given for each tested EPS product in the TDS, was compared with the corrected resistance *R*(*d*). The requirements of Standards [31,32] were satisfied.

The *R*(*d*) values for the EPS A and B panels were calculated by converging the corrected *λ*(*d*) data (see Supplementary Material part 1). Yet, for the EPS C panels, the *R*(*d*) were directly assigned to the *R*′(*d*), as measured on the HFM Fox 600 (see Section 2.4.3).

**Figure 5:**
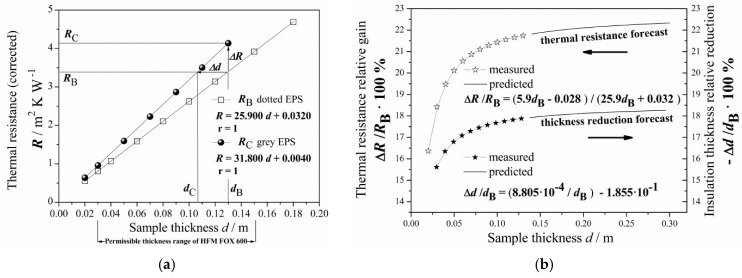
(**a**) The thermal resistance, *R*, at Tm = 10 °C versus the panel thickness *d* for the EPS B and C products. The equations resulting from the linear fit (1.9) from Supplementary Material part 1 are shown together with the linear correlation coefficient r (solid lines). Δ*R* shows the difference between B and C panels of the same thickness and Δ*d* shows the difference between the B and C panels of the same *R*-value. (**b**) The thermal resistance relative gain Δ*R/RB* (left axis) and the insulation thickness relative reduction −Δ*d/dB* (right axis) for EPS C with respect to EPS B, versus the panel thickness *d*. The graph is reflected horizontally, since Equation (8) gives negative values. The experimental points are extrapolated (solid lines) based on the functions shown, corresponding to Equations (9) and (11).

In Figure 5a, only the EPS B and C products were compared, as the EPS A and B panels differed too much in average bulk density (Table 2), which made their comparison not precise. In EPS C, resistance increased faster with thickness. Thus, considerable improvement in thermal insulation performance appears evident, when comparing the *R*-values of “grey” EPS C to “dotted” EPS B.

To account for the data, the linear model (1.9), from Supplementary Material part 1, was applied to the *R*(*d*) points. After the linear fit, in order to examine the effect of GMP, Δ*R* was defined as the difference between the EPS B and C panel’s *R*-values at a given thickness *d*_B_:

(5) $$\Delta R=R_{C}-R_{B}$$

This is shown as a vertical arrow, Δ*R*, in Figure 5a. Hence, the relative gain Δ*R*/*R*_B_ in thermal resistance of the EPS C, with respect to EPS B of the same panel thickness *d*, could be defined as:

(6) $$\frac{\Delta R}{R_{B}}=\frac{R_{C}-R_{B}}{R_{B}}$$

which is plotted in Figure 5b as a function of *d*.

Maintaining a constant given thermal resistance level, the corresponding change in the insulating layer thickness could be defined, for the EPS B and C panels, by:

(7) $$\Delta d=d_{C}-d_{B}$$

which is shown as a horizontal arrow, Δ*d*, in Figure 5a. As can be seen, greater the thermal resistance *R*(*d*), the greater is the difference in thickness Δ*d*. Hence, the relative change Δ*d*/*d*_B_ in thickness of the EPS C, with respect to EPS B of the same *R*-value, can be defined as:

(8) $$\frac{\Delta d}{d_{B}}=\frac{d_{C}-d_{B}}{d_{B}}$$

which is also plotted in Figure 5b as a function of *d*.

From Equation (6) and the system of equations (Figure 5a), one may derive the analytical expression for the percentage increase (gain) in thermal resistance:

(9) $$\frac{\Delta R}{R_{B}}\cdot 100\%=\frac{5.9d-0.028}{25.9d+0.032}\cdot 100\%$$

where *d* = *d*_B_ = *d*_C_ may range from 4.7·10^−3^ to 0.30 m. From Equation (9), one may calculate the maximum asymptotic value of about 22.8% (as *d*_B_ → ∞). In practice, it is possible to achieve the maximum value of 22.3% only, for panels of the highest available thickness (0.30 m).

By using the linear fit equations reported in Figure 5a, one may find the practical equation:

(10) $$d_{C}=0.814d_{B}+0.0008805$$

where *d*_B_ is the thickness (in m) of EPS B and *d*_C_ is the thickness of the EPS C panel of the same *R*-value (*R*_B_ = *R*_C_).

From Equations (7) and (10), one may derive the analytical expression for the percentage change in thickness:

(11) $$\frac{\Delta d}{d_{B}}\cdot 100\%=\left(\frac{0.0008805}{d_{B}}-0.1855\right)\cdot 100\%$$

which is valid for *d*_B_ ranging from 4.7 × 10^−3^ to 0.30 m. Expression (11) allows negative values; thus, the magnitude of thickness reduction increases with the insulation thickness, *d*_B_. The thicker the insulating layer required, the greater is the benefit in terms of material and cost savings when replacing EPS B with EPS C. As calculated from Equation (11), the outermost theoretical value of the percentage change could be achieved as −18.55% (as *d* → ∞). In practice, it is possible to get only −18.26% for panels of the highest available thickness (0.30 m).

Finally, based on Equations (9) and (11), the additional analytical expression relating the thickness reduction and the resistance gain can be rewritten as:

(12) $$-\frac{\Delta d}{d_{B}}=\frac{0.001006}{d_{B}}\frac{\Delta R}{R_{B}}+0.8145$$

It is worthy to highlight that |−Δ*d*/*d*_B_| ≠ |Δ*R*/*R*_B_|, so the relative resistance gain does not directly determine the relative thickness reduction, especially for thicker EPS panels. If *d* = 0.30 m, then −Δ*d*/*d*_B_ ≈ 0.82 Δ*R*/*R*_B_. In other words, when considering EPS B and C, the relative thickness reduction |−Δ*d*/*d*_B_| may reach only 82% of the relative resistance gain, Δ*R*/*R*_B_, when increasing thickness up to 0.300 m. When decreasing the EPS B panel thickness, this relation achieves equivalence, such that −Δ*d*/*d*_B_ ≈ 1.00 Δ*R*/*R*_B_ at the theoretical value *d* ≈ 0.005 m.

### 3.5. Micro-Raman Spectra of Tested Products

The Raman spectra were separately collected for the “white” part selected only from the “dotted” EPS B and for the “grey” EPS C products. The “white” part (no black dotted isles) cut from the EPS B, one may consider as the equivalent of “white” EPS material (pure). Raman spectra were registered for two excitation wavelengths to distinguish phonon and luminescent peaks.

**Figure 6:**
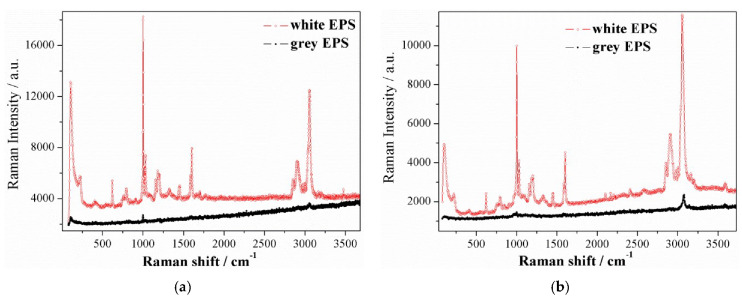
(**a**) Raman spectra for selected “white” part of the EPS B (red line) and for the “grey” EPS C (black line) products. The 633 nm He–Ne-ion laser line was used as the excitation wavelength. (**b**) Raman spectra for selected “white” part of the EPS B (red line) and for the “grey” EPS C (black line) products. The 514 nm Ar-ion laser line was used as the excitation wavelength.

According to the above indicated, for the “white” EPS material (red lines in Figure 6a,b), without GMP, in both Raman spectra, the bands characteristic for the pure polystyrene matrix were observed. There are intensive phonon modes related to phenyl ring: ~650 cm^−1^, ~1100 cm^−1^, and ~1600 cm^−1^ and hydrocarbon chain modes in the range of 2900–3000 cm^−1^ [56,57]. In low frequencies, below 200 cm^−1^, an increasing band corresponding to the Boson peak was detected [58]. The bosonic peak is derived from acoustic phonons and appears in glasses and amorphous materials, where the selection rules for Raman scattering have been broken. It is observed as low-shaped, often asymmetrical, broad peak occurring in the low frequency region of the Raman spectrum (below 200 cm^−1^).

For the “grey” EPS C (black lines in Figure 6a,b), in both Raman spectra, a significant decrease in Raman spectral intensity was observed. Such suppression of phonon spectra is usually associated with structural deformation of the molecules or molecule chains, which results in transitions to more disordered structural forms of polymer matrix [59]. The suppression effect was observed for both optical and acoustic phonons for the polystyrene matrix with GMP. The attenuation of acoustic phonons for the polystyrene matrix with GMP is indirectly visible due to the absence of boson peak in the Raman spectrum. In polymeric insulation where there are no free to move carriers, the polymer matrix thermal conduction is determined by phonons, especially by acoustic phonons. The fact that acoustic and optical phonons are suppressed in the matrix with GMP results in lowering the matrix thermal conduction and, thus, the total thermal conductivity of the EPS C insulation.

Moreover, the Raman spectra of the “grey” EPS C show a decrease in the background signal, resulting from luminescence. Such a process may also indicate an increased electromagnetic radiation absorption coefficient for graphite-containing EPS insulation.

It should be emphasized that Raman studies for the “grey” EPS C do not show graphite-specific peaks. This is different than in the case of carbon nanotubes-containing polystyrene samples [60]. Therefore, the addition of GMP to the polystyrene matrix results in modified/disturbed matrix and the EPS C cannot be treated as a simple mixture of polystyrene and graphite.

### 3.6. Thermal Analysis of Tested Products

The TGA and DSC thermograms were separately collected for the “white” part selected only from the “dotted” EPS B and for the “grey” EPS C products. One may consider the “white” part (no black dotted isles) as the equivalent of “white” EPS material (pure).

**Figure 7:**
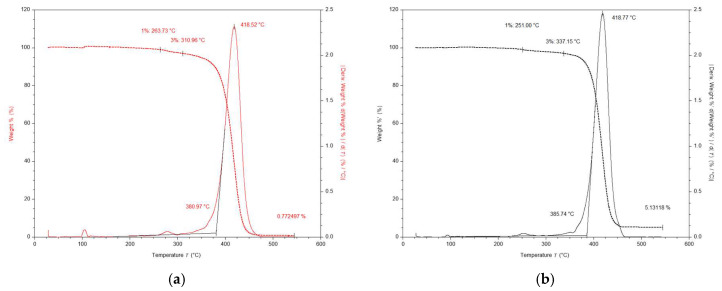
TGA thermograms for: (**a**) selected “white” part of the EPS B (red lines) and (**b**) the “grey” EPS C (black lines) products.

According to the above indicated data, the TGA thermograms indicate an improvement on the thermal stability of the “grey” EPS C compared with the “white” EPS material, as shown in Figure 7a,b. The initial mass losses of 3% occurred as follows: 311 °C for the “white” EPS, 337 °C for the EPS C, in the insulation samples with mass ranging between 1.994 and 1.968 mg, respectively. One may attribute the considerable increase in thermal stability of the EPS C to homogenous GMP dispersion in polystyrene matrix. Additionally, the presence of GMP impedes the burning process by reducing the oxygen diffusion towards bulk. The resulting maximum degradation rates (calculated from percentage mass change derivative) were as follows: 418.5 °C for the “white” EPS and 418.8 °C for the EPS C samples. The mass loss of the insulation samples occurred at only one stage and finally reached at 550 °C as follows: 0.8% and 5.1%, respectively. Thus, the graphite content calculated as the difference between the EPS C and “white” residue masses, 0.100 mg and 0.016 mg respectively, was up to 4.3% of the total mass of the EPS C sample.

During the first conventional DSC heating scan, the resulting midpoint of *T*_g_ was as follows: 107.8 °C for the “white” EPS material and 109.7 °C for the “grey” EPS C, in the insulation samples with mass ranging between 2.274 and 2.248 mg, respectively. The melting temperature (*T*_cm_), typical for the crystalline phase, was not observed in the EPS C, even up to the degradation temperature occurring at 350 °C (see degradation onsets at 381 °C and 386 °C in Figure 7). In the case of the “white” EPS, one may detect a small exothermic peak due to the cold crystallization at 194.5 °C and the melting peak at *T*_cm_ = 282.9 °C; yet, the calculated crystallinity *X_C_* = 0.7%, was extremely low. The increase in *T*_g_ of the EPS C can be explained, as the effect of intermolecular interactions between GMP and the closest polystyrene matrix chains, thereby reducing the mobility of the polymer chains and thus increasing the *T*_g_ value.

## 4. Discussion

### 4.1. Thermal Conductivity Analysis

#### 4.1.1. Relationship between Thickness Effect and Density

For industrially produced “pure” EPS panels, the literature has reported that bulk density is the dominant controlling variable determining the mechanical and thermal properties [54]. The *λ*(*ρ*) function has been observed in the range of 10–45 kg·m^−3^, where total thermal conductivity decreased with increasing bulk density [6,27,37,41,43,54]. In particular, the coefficient *λ* can slightly decrease from 0.041 to 0.038 W·m^−1^·K^−1^, whereas bulk density increases from 16 to 18 kg·m^−3^. The average value <*λ*_A_> ≈ 0.039 W·m^−1^·K^−1^ at <*ρ*_A_> ≈ 17 kg·m^−3^ found in this study was in agreement with the literature [37,43]. Furthermore, the average values for EPS B and C agreed with the literature as well [42].

Comparing Figure 2 and Figure 3, one may observe the impact of varying bulk density gradients on thermal conductivity changing along thickness. The density function *ρ*_A_(*d*), visibly “waving” around its average level, <*ρ*_A_>, seems to be synchronized with conductivity function, *λ*_A_(*d*), which simultaneously “waves” around its average level, <*λ*_A_>, yet, opposite in phase (Figure 4b). In the case of EPS B and C, *λ*_B_(*d*) and *λ*_C_(*d*) did not display such unsteadiness; instead, these materials revealed more uniform packing of the EPS beads or better structural homogeneity (in terms of cell morphology in the beads). These effects are understandable as, in practice, thermal conductivity (either corrected or not) is the composite function *λ* of *ρ* versus *d*, such that:

(13) λ(ρ,d)=(λ∘ρ)(d)=λ(ρ(d))

As reported in [37,43], the thickness effect is much more visible for very low bulk density “pure” EPS than in greater density panels. The more significant the effect, the longer the curvature and the further the thickness limit position *d*_L_, the value of which decreases as the bulk density increases. The results for the EPS A and B panels were in good agreement with the literature, as *d*_LA_ < *d*_LB_ while *ρ*_A_ > *ρ*_B_ (Table 2 and Table 3). Yet, unlike the results explained in [37], the thickness effect may not have originated from the experimental setup. According to [37], radiation can be blocked by reflection from the colder black plate and might not be absorbed by a thinner “pure” EPS panel before returning to the “hot” black plate; this was expected to reduce the electric power required by the heating system in HFM and, hence, lower measured conductivity of the thermal insulation. Alternatively, in the light of the relation (13), the thickness effect could be explained as a simple consequence of the structural differences between the rough sample surface region (EPS panel interfacing with the “hot” or “cold” HFM black plate) and the deeper bulk core region (of slightly lower bulk density). Thus, this effect, which is common in lighter EPS products, may result from the density gradient (normal to the panel surface). Moreover, it is due to conduction (of the matrix component), rather than radiation, which is shown in Section 4.1.3.

#### 4.1.2. GMP Effect

Another outcome from the literature is that, when comparing EPS foams with and without GMP, the nearly constant levels reached by *λ*′(*d*) (as in Figure 4a) differ; furthermore, this difference is greater with lower EPS density [37,43]. Dependence on GMP is also evident from Figure 4b, where the <*λ*> level drops gradually with increasing GMP content. To understand the effect of GMP on the total thermal conductivity levels, one may look at the impact of GMP on the individual components of total thermal conductivity, which can be assumed to be additive. As air cannot flow through the EPS closed-cell structure, the convection component can be neglected in this particular case [1,27]. Therefore, total thermal conductivity of the EPS should be resolved into its three main components:

- radiation (through both solid matrix and air),
- solid matrix conduction, and
- gas conduction (air thermal conductivity without radiation).

**Figure 8:**
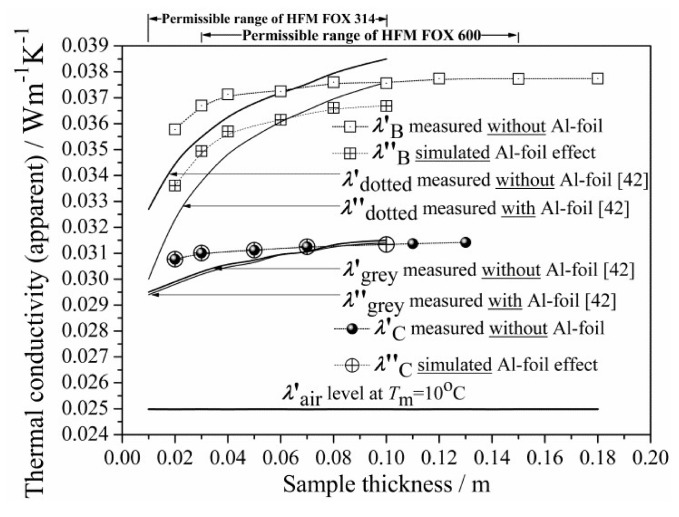
The effects of Al-foil and GMP on the thermal conductivity of EPS. The dotted squares and black spheres show the apparent thermal conductivity *λ*′(*d*) at *T*_m_ = 10 °C for EPS B and C, respectively. The crossed squares and circles show the simulated data of *λ*″(*d*) for EPS B and C with Al-foil. The solid lines are plotted based on data from [42]. The thin and thick lines indicate tests with and without Al-foil, respectively.

To this end, an interesting analysis was done by compilation and comparison of the experimental results and data reported in the literature [42] (indicated in Table 4), by combining the HFM Fox 600 and Fox 314 measurements (with and without the two parallel 10 µm Al-foil layers at the bottom and on the top of the sample). All EPS products were of comparable, very low bulk density, from 14.0 to 15.0 kg·m^−3^. Figure 8 presents the combined data, together with an additionally simulated Al-foil effect on EPS B and C.

**Figure 9:**
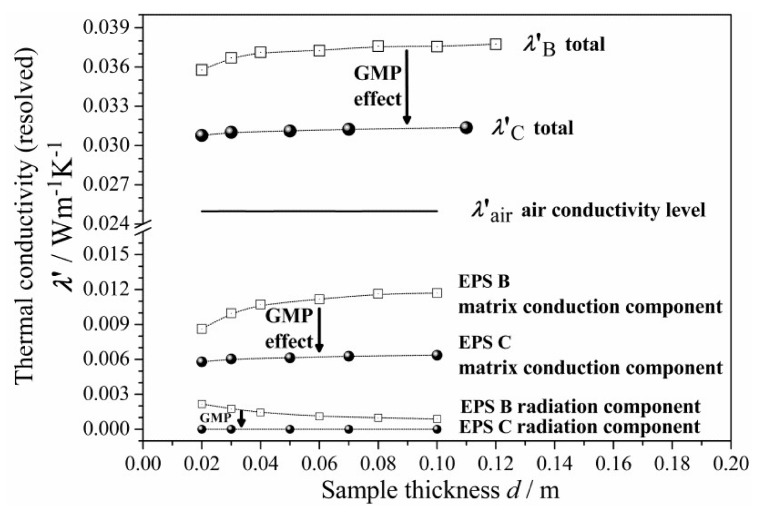
The GMP effect on the total thermal conductivity, resolved into its components. Each component value is calculated by multiplying the contribution fraction (Table 5) by the total thermal conductivity. Notice the impact of GMP on the thickness effect.

Based on this data set (Table 4) and the collected curves (Figure 8), one could carry out the quantitative estimation of each thermal component contribution by applying the procedure described in Supplementary Material part 3. The calculated results, in terms of percentage contributions, are listed in Table 5 (for the first time) as well as visualized in Figure 9 for the tested EPS B and C, in terms of the resolved components of total thermal conductivity.

As can be seen from Table 5, the numerical change of each component (radiation, air conduction, and matrix conduction) could be best observed for thicknesses from 0.01 to 0.10 m, simultaneously for the two GMP industrial concentrations (EPS B and C tested or the corresponding EPS “dotted” and “grey” from [42]). For thickness up to 0.10 m, the percentage contributions are shown, which describe the evolution of all components of total thermal conductivity (before and after correction) versus thickness and GMP content.

As one may notice from Table 5, the radiation contribution to the total thermal conductivity of the “dotted” EPS from [42] decreased from 8.3% to 2.3%, whereas the matrix conduction contribution increased from 15.3% to 32.8%, with thickness increasing from 0.01 to 0.10 m. Comparing the obtained percentage contribution at each thickness, it appears that the matrix conduction may play a greater role than the radiation in the overall heat transport through EPS. For the “dotted” EPS panels of the lowest thickness, the radiation contribution appeared to be no more than 8.3%, whereas the matrix contribution result was 15.3%—nearly twice as big. On the other hand, in the thicker 0.10 m EPS panels, the radiation contribution dropped down to 2.3% and the matrix contribution reached 32.8%, such that the thicker sample had a much greater matrix contribution. For the EPS B tested panels, similar trends can be observed.

For the “grey” EPS from [42], the radiation contribution was zero and the matrix contribution revealed an increasing trend, from 15.3% to 20.7% (at the expense of the air contribution), with thickness increasing from 0.01 to 0.10 m. For EPS C, the radiation contribution was also zero and the matrix contribution revealed a similar increasing trend—from 18.8% to 20.5%—with thickness increasing from 0.02 to 0.11 m. For the EPS C tested panels, similar trends can be observed.

Thus, comparing from Table 5 (as mentioned above), the calculated results for the corresponding EPS “dotted” and “grey” from [42] with EPS B and C, one may notice that there was a good agreement, in terms of the observed trends and the calculated values.

Interestingly, it may be noticed from Figure 8 that, after applying Al-foil, the *λ*″_dotted_ (or *λ*″_B_) did not drop down to the *λ*″_grey_ (or *λ*″_C_) level for any *d*. In order to explain the apparent gap between the “grey” and “dotted” EPS material’s, i.e., the total thermal conductivity levels (both with Al-foil and thus, without radiation), one must take into account the polymer matrix conduction component, which, besides radiation, can also be reduced by the GMP. The latter fact seems to have been neglected in the literature [42,43,44,45,46,47,48,49,50]. One may try to explain that the *λ*″_dotted_ (or *λ*″_B_) may not have dropped more, due to insufficient blocking (cutting off) of radiation by the Al-foil or by some “secondary” radiation generated internally (some flux of the phonons could be converted into photons). Yet, one must notice that the Al-foil emissivity value is no more than 0.04 and that above 96% of the photon flux was reflected back by the upper Al-foil, either “primary” or “secondary”. Hence, the concept of such “secondary” radiation cannot explain such an evident gap. Facing the facts that radiation can be efficiently blocked by reflection from the Al-foil and that the conductivity gap appears, the effect of GMP on the polymer matrix conduction becomes evident; that is, after addition of the GMP, conduction of the polymer matrix is dramatically reduced. The latter effect is also visualized in Figure 9. In particular, the absolute negative change in the matrix conductive component (e.g., from about 0.012 down to 0.006 W·m^−1^·K^−1^ at 0.10 m) can be one order of magnitude greater than the radiative one (from about 0.001 down to 0.000 W·m^−1^·K^−1^ at 0.10 m).

As observed in Figure 9, the total thermal conductivity of the “grey” EPS C, relative to the “dotted” EPS B, was reduced, which might be due to both the thermal radiation (through the whole foam) drop and conduction (only through the polymer matrix with graphite, not air) drop. This might be caused by several physical phenomena. In particular, the interfacial effects that are induced by incorporation of the GMP at the highest concentration could be responsible [61,62,63,64]. From a microscopic view, GMP is a specific opacifier of proper size (to prevent agglomeration), evenly distributed in the polymer matrix and located between the solid (matrix) and gas (air) phase, hence forming additional interfaces (GMP/air and GMP/matrix).

A question which arose was: why does GMP cause such a dramatic decrease in the thermal conduction component of the polymer matrix, the lower-than-expected, when comparing the conduction to radiation drop, observed in Figure 9, for both EPS-GMP industrial systems? First, phonons are hindered at the GMP/air and matrix/GMP interfaces as well as at the GMP exterior and interior regions. At the interfaces, they are either (strongly) scattered or (much less probable) blocked by reflection on GMP. The presence of various scattering processes for phonons leads to a reduction in their lifetime and thus, also to slow down the heat transport process taking place with their participation. At the GMP exterior region, phonons can also be temporary blocked or delayed, due to the intermolecular interactions between GMP and the closest polystyrene matrix chains causing modified/disturbed matrix, as it results from the increase of *T*_g_ value (Section 3.6) and supressed Raman spectra (Figure 6). At the GMP interior region of high thermal capacity, further (strong) delay is caused by absorption–emission in random direction (by the delocalized nonbonded electrons of the sp^2^ carbon atoms in GMP), simultaneously to (rare) refraction of the phonons. The above may produce a local thermally isolative barrier, increasing resistance. As a result, the presence of GMP significantly improves the insulating qualities of EPS materials.

#### 4.1.3. Further Explanation of the Thickness Effect

As mentioned above, the thickness effect can be related to bulk density [37,43], however, it is also related to the GMP concentration changes. As shown in Figure 4a, the results for the EPS B and C were in good agreement with the literature, as *d*_LC_ < *d*_LB_, while the GMP concentration was greater in EPS C than in EPS B at comparable bulk densities. The maximum of *λ*′_C_(*d*) differed from the minimum value by only 2%, which indicates a dramatic reduction in the observable thickness effect due to the addition of GMP. Other researchers have also found a negligible thickness effect in such “grey” EPS C-like products [37,42,43].

On one hand, the thermal conductivity of “grey” EPS, *λ*′_grey_ (or *λ*′_C_), almost does not depend on thickness and, thus, practically does not reveal the thickness effect, whereas the conductivity of “dotted” EPS, *λ*″_dotted_ (or *λ*″_B_), even measured with Al-foil, still does cutting off radiation by Al-foil did not remove the conductivity curvature (compare the curves for “dotted” EPS in Figure 8). Thus, distributing GMP with a higher industrial concentration in the polymer matrix may significantly reduce the thickness effect, as compared to the “pure” or “dotted” EPS with or without Al-foil.

On the other hand, applying Al-foil to the “grey” EPS practically did not cause any further decrease in the measured thermal conductivity *λ*′_grey_ (Figure 8). Therefore, GMP and Al-foil seem to have a similar effect in terms of blocking the thermal radiation flux.

Besides the relation between density and the thickness effect, the above observations from the experiment with Al-foil (which effectively blocks radiation though permitting conduction) suggest that the thickness effect is caused by matrix conduction (increasing with depth when crossing the EPS surface region of higher density) rather than radiation.

Thus, both observed phenomena—reduction of the thickness effect and the significant drop of total thermal conductivity after addition of GMP—might be caused by a stronger decrease in polymer matrix conduction (e.g., from about 0.012 to 0.006 W·m^−1^·K^−1^ at 0.10 m) than a decrease in the thermal radiation component (0.001 to 0.000 W·m^−1^·K^−1^ at 0.10 m, based on Table 5 and Figure 9). Note that radiation reveals a very poor contribution at the applied temperature difference.

Comparing the curves in Figure 9, the matrix conduction clearly reveals responsibility for the observed thickness effect on the EPS thermal conductivity (i.e., the *λ*′_B_ total and the EPS B matrix conduction are convex, while the EPS B radiation component is concave). As also seen in Figure 9, the EPS C matrix conduction component does not reveal the thickness effect at all. By this feature, one may discover that GMP manifests a strong effect on the polymer matrix. This must be stronger than the effect of the density gradient (normal to surface) as well as stronger than interfacial effect at the EPS panel rough surface (matrix/air).

## 5. Conclusions and Evaluation

This study analyses heat transfer in practical closed-cell EPSs insulation. Some conclusions and evaluation derived from the experimental and simulated findings are summarized below.

1. Initial testing of EPS product quality should be a homogeneity assessment, which can be based either on bulk density or thermal conductivity measurements versus thickness. This is possible due to the observed *ρ*(*d*) and corrected *λ*(*d*) synchronizing and the experimental relationship of *λ(ρ*) between the density and corrected conductivity functions. Absence of data scattering and constant level indicates good quality. The worst homogeneity was found for the “white” EPS A product of poor quality, possibly due to recycling process used during production. The “dotted” EPS B and “grey” C products revealed good homogeneity. As the poor homogeneity may have a great impact on all thermal measurements and material characteristics, the EPS A product had to be excluded from further analysis.
2. The analysis and evolution of the total thermal conductivity components versus the EPS panel thickness in the range of 0.02–0.1 m, for two different GMP concentrations (which are applied industrially): low (“dotted”) and high (“grey”) were described. The EPS materials from which the panels were made had comparable and very low bulk densities, from 14 to 15 kg·m^−3^. The simulated data for the “dotted” EPS B and “grey” EPS C products are presented in Table 5 and plotted in Figure 8. The analysis was carried out by combining experimental measurements (HFM Fox 600) and literature data (HFM Fox 314) [42]. Simulation of the thermal radiation component was carried out through the above data processing, which was used to separate all thermal conductivity components (radiation, air conduction, and matrix conduction), as plotted in Figure 9. A lack of convection was assumed, due to the EPS closed-cell structure. The percentage contributions of all thermal components were then calculated.
3. In EPS materials that differ in GMP concentration (“dotted” and “grey”), the percentage contribution of the polymer matrix thermal conduction component and the thermal radiation component in the total thermal conductivity vary with the thickness of the thermal insulating layer in both product types. In detail, we noticed the following main points (Table 5):
  - In the “dotted” EPS B at the smallest panel thickness (up to the thickness limit value), the thermal radiation component reached its highest percentage contribution in the total heat transport. At the smallest thickness (of 0.02 m), the thermal radiation contribution was 10.8% (corrected data in brackets). The thermal radiation contribution decreased with an increase of the panel thickness. Above the thickness limit, the contribution of the thermal radiation component was negligible and, at the highest available thickness of 0.1 m, it was only 2.3% (corrected data in brackets). On the contrary, the contribution of the solid matrix thermal conduction component increased with an increase of panel thickness. The contributions of the polymer matrix thermal conduction component were 22.9% and 31.2% (corrected data in brackets) for the 0.02 and 0.1 m panels, respectively; and
  - In the “grey” EPS C, regardless of the panel thickness, the thermal radiation component was negligible. The percentage contribution of the polymer matrix thermal conduction component was 18.8% in the 0.02 m panels. The contribution increased with thickness and reached 20.4% at the highest panel thickness (0.1 m).
4. As resulted, adding GMP in high industrial concentrations as in “grey” EPS material may force a change in the radiative–conductive heat transfer mechanism; yet, it does not cause a perceivable decrease of the air conduction contribution. Based on the analysis results presented in Table 5, unfortunately, the percentage contributions in both the “dotted” EPS B and “grey” C products at the smallest panel thickness (0.02 m) can reach up to 70% and 81% and at the highest panel thickness (0.1 m), to about 66% and 80%, respectively. In order to reduce air conduction contribution, one may apply volume compression during foam manufacturing, as in the case of XPS foam production [65]. Such volume compression may be realized in combination with cell morphology regulation by altering the cell orientation (in one preferred spatial direction) and cell anisotropy (of 3D form), as compared with substantially round celled materials [66]. Additionally, one may reduce the cell size to obtain nanocellular PS foams [67,68].
5. The comparison of EPS materials (“dotted” and “grey”), regarding their distributions of percentage contributions of thermal conductivity components (Table 5, e.g., the “dotted” EPS B and “grey” C products) at the highest panel thickness (0.10 m), showed a dramatic effect of change in thermal radiation, by nearly −100% (i.e., (0–0.023)/0.023 × 100%). Furthermore, the polymer matrix thermal conduction was reduced strongly, by c.a. 35% (i.e., (0.203–0.312)/0.312 × 100%). One may conclude that the incorporation of GMP implicates elimination of the thermal radiation. It also considerably weakens the polymer matrix thermal conduction, especially for large thickness panels, as the contribution of the matrix conduction becomes substantial for panels above the thickness limit. In general, the results indicate that the higher the thickness, the greater the reduction effect of matrix thermal conduction.
6. The apparent evolution of all thermal conductivity components was found in the analysis, based on measured and simulated data for EPS materials of two different GMP industrial concentrations (“dotted” and “grey”). In order to confirm the observed effects, verification may be required in terms of additional measurements. Yet, the trends revealed in this experiment are not expected to radically change.
7. As shown in Figure 6, the GMP addition to the polystyrene matrix (as in “grey” EPS C) leads to polymer matrix structural modification processes, resulting in significant attenuation of phonon spectra characteristic of pure matrix (as in “white” EPS material). This directly supports the observed drop in matrix thermal conduction component (Figure 9) and thereby explains the decrease in total thermal conductivity of EPS insulation (Figure 8). It is well known that the graphite’s thermal conductivity is very high. However, based on Raman spectra, we can conclude that the addition of GMP does not lead to a simple mixture of graphite and polystyrene. In the Raman spectrum of the matrix of the EPS C, there are no modes characteristic for graphite. It should be assumed that we are dealing with particular intermolecular interactions between graphite particles and polystyrene, leading to a structurally modified/disturbed polymer matrix.
8. The thermal isolation of required resistance can be designed, regarding EPS “grey”, rather than EPS “dotted” or EPS “white” panels, of reduced thickness (0.18–0.30 m) and at comparable density to EPS materials. In building practice, this means that the highest achievable reduction of at least 18.3% in the EPS insulating layer thickness is possible, referring to the thickest 0.30 m “dotted” EPS B or even “white” EPS panels.

## Figures and Tables

**Table 1:** Data concerning the Standard Reference Material (SRM) sample (size, bulk density, thermal conductivity, and the correction parameter).

**SRM Type**		**SRM Dimensions (m)**				**SRM Bulk Density *ρ*_SRM_ (kg·m^−3^) Average Value given in the Certificate**	
		**Length *x***	**Width *y***		**Thickness *d***		
Glass wool IRMM-440		0.500	0.500		0.0347	75.8	
**SRM thermal conductivity coefficients at** ***T*_m_ = 10 °C (W·m^−1^·K^−1^)**				**Correction parameter *𝒞* at the coverage factor k = 2.0 *𝒞* = *λ*^C^_SRM_ − *λ*^M^_SRM_ (W·m^−1^·K^−1^)**		**Expanded uncertainties (W·m^−1^·K^−1^)**	
***λ*^C^_SRM_ Average value given in the Certificate**	***λ*^M^_SRM_ Average value measured by HFM FOX 600**					***U*(*λ*^C^_SRM_)**	***U*(*λ*^M^_SRM_)**
0.03048	0.03057			−9 × 10^−5^		2.8 × 10^−4^	3.2 × 10^−4^

**Table 2:** Data concerning density measurements of the materials expanded polystyrene (EPS) A, B, and C.

EPS Type	Panels Mass Range (kg·10^−3^)	Panels Dimensions (m)			Bulk Density (kg·m^−3^)			Average Expanded Uncertainty <*U*(*ρ*)> (kg·m^−3^)
					<*ρ*_A_>	<*ρ*_B_>	<*ρ*_C_>	
		Length *x*	Width *y*	Thickness Range *d*	Average Value with Double Standard Deviation ±2σ			
A “white”	103.14–756.90	0.60	0.50	0.02–0.15	16.9 ± 1.2			2.1 × 10^−1^
B “dotted”	87.06–666.00	0.60	0.50	0.02–0.18		14.2 ± 0.2		1.8 × 10^−1^
C “grey”	90.48–567.84	0.60	0.50	0.02–0.13			14.6 ± 0.5	2.2 × 10^−1^

**Table 3:** Data concerning the thermal conductivity of the EPS A, B, and C products (thermal transmissivity declared and averaged coefficients, with corresponding thickness limits).

EPS Type	Thickness Limit *d*_L_ (m)	Thermal Transmissivity *λ*_t_ (W·m^−1^·K^−1^)	Thermal Conductivity Coefficients at *T_m_* = 10 °C (W·m^−1^·K^−1^)				Average Expanded Uncertainty <*U*(*λ*)> (W·m^−1^·K^−1^)
			*λ*_D_	<*λ*_A_>	<*λ*_B_>	<*λ*_C_>	
				Average Value with Double Standard Deviation ±2σ			
A “white”	0.043 ± 0.010	0.0400	0.040	0.0394 ± 0.0010			1 × 10^−3^
B “dotted”	0.060 ± 0.005	0.0386	0.040		0.0377 ± 0.0001		1 × 10^−3^
C “grey”	0.007 ± 0.001	0.0314	0.032			0.0312 ± 0.0005	8 × 10^−4^

**Table 4:** A brief comparison of EPS B and C (tested) with the corresponding EPS (“dotted” and “grey”) from [42] (marked as literature data) and the list of HFM instruments with test setup and output data.

**EPS Materials**	**Low Industrial Concentration of GMP in the “dotted” EPS Materials**			
	**“dotted” EPS (Adapted)—Literature Data [42]**		**“dotted” EPS B (Tested)—Measured and Simulated Data**	
HFM instrument and test setup	HFM FOX 314		HFM FOX 600	
	Without Al-foil	With Al-foil	Without Al-foil	With Al-foil
	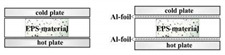		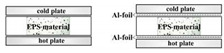	
HFM output as the apparent thermal conductivity coefficient	Measured *λ*′_dotted_ (*d*)	Measured *λ*″_dotted_ (*d*)	Measured *λ*′_B_ (*d*)	Simulated *λ*″_B_ (*d*)
**EPS Materials**	**High Industrial Concentration of GMP in the “grey” EPS Materials**			
	**“grey” EPS (Adapted)—Literature Data [42]**		**“grey” EPS C (Tested)—Measured and Simulated Data**	
HFM instrument and test setup	HFM FOX 314		HFM FOX 600	
	Without Al-foil	With Al-foil	Without Al-foil	With Al-foil
	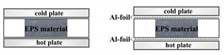		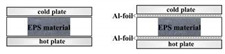	
HFM output as the apparent thermal conductivity coefficient	Measured *λ*′_grey_ (*d*)	Measured *λ*″_grey_ (*d*)	Measured *λ*′_C_ (*d*)	Simulated *λ*″_C_ (*d*)

**Table 5:** Percentage contributions of the total thermal conductivity components (radiation, air conduction, and polymer matrix conduction) presented for the apparent λ′(d) and corrected λ(d) (in brackets). The calculated results compare EPS B and C (tested) with the corresponding EPS (“dotted” and “grey”) from [42].

		Radiation	Air Conduction	Polymer Matrix Conduction	Radiation	Air Conduction	Polymer Matrix Conduction
		Contribution (%)			Contribution (%)		
**Thickness (m)**		**Low Industrial Concentration of GMP in the “dotted” EPS Materials**					
		**“dotted” EPS (adapted)—Based on Literature Data [42]**			**“dotted” EPS B (tested)—Based on Measured and Simulated Data (in Brackets Corrected below *d*_LB_ = 0.060 m)**		
0.01		8.3	76.4	15.3	-	-	-
0.02		6.1	72.2	21.7	6.1 (10.8)	69.8 (66.3)	24.1 (22.9)
0.03		4.8	70.4	24.8	4.8 (7.4)	68.1 (66.2)	27.1 (26.4)
0.04		3.9	68.8	27.3	3.9 (5.4)	67.3 (66.2)	28.8 (28.4)
0.05		3.5	67.9	28.6	-	-	-
0.06		3.0	67.1	29.9	3.0 (3.9)	67.1 (66.4)	29.9 (29.7)
0.07		2.7	66.6	30.7	-	-	-
0.08		2.6	65.7	31.7	2.6 (2.6)	66.4 (66.4)	31.0 (31.0)
0.09		-	-	-	-	-	-
0.10		2.3	64.9	32.8	2.3 (2.3)	66.5 (66.5)	31.2 (31.2)
0.11		-	-	-	-	-	-
**Thickness (m)**		**High Industrial Concentration of GMP in the “grey” EPS Materials**					
		**“grey” EPS (Adapted)—Calculated Based on Literature Data [42]**			**“grey” EPS C (Tested)—Calculated Based on Measured and Simulated Data**		
0.01		0	84.7	15.3	0	–	–
0.02		0	83.5	16.5	0	81.2	18.8
0.03		0	82.4	17.6	0	80.6	19.4
0.04		0	81.6	18.4	0	–	–
0.05		0	81.4	18.6	0	80.3	19.7
0.06		0	80.6	19.4	0	–	–
0.07		0	80.6	19.4	0	79.9	20.1
0.08		0	79.6	20.4	–	–	–
0.09		–	–	–	–	–	–
0.10		0	79.3	20.7	0	≈ 79.7	≈ 20.3
0.11		–	–	–	0	79.6	20.4

## Supplementary Materials

The following are available online at https://www.mdpi.com/1996-1944/13/11/2626/s1.

## The List of Symbols and Abbreviations

Symbols and Abbreviations	Meanings	Units
<…>	– distinguishing symbol for a physical quantity averaged over thickness range	
A	– “white” (pure) EPS product, tested herein or elsewhere in literature	
B	– “dotted” EPS product, tested herein	
dotted	– “dotted” EPS product, tested elsewhere [42]	
C	– “grey” EPS product, tested herein	
grey	– “grey” EPS product, tested elsewhere [42]	
*𝒞*	– correction parameter (Table 1)	(W·m^−1^·K^−1^)
CEN	– European Committee for Standardization	
DSC	– differential scanning calorimetry	
TGA	– thermogravimetric analysis	
d	– thickness (height) of the panel or sample	(m)
*d*_A_, *d*_B_, *d*_C_	– thickness of the panel or sample referring to product A, B, or C, respectively	
*d*_max_	– maximum permissible thickness of the sample assigned to the chamber of specified dimensions of the plate instrument	(m)
*d*_min_	– minimum permissible thickness of the sample assigned to the chamber of specified dimensions of the plate instrument	(m)
*d*_L_	– the panel thickness limit of the *R*′ and *λ*′ (non-)linearity	(m)
*d*_LA_, *d*_LB_, *d*_LC_	– the thickness limit referring to product A, B, or C, respectively	
EPS/XPS	– expanded/extruded polystyrene	
GFMs	– gas-filled materials	
GHP	– guarded hot plate instrument	
GMP	– graphite microparticles	
HFM	– heat flow meter instrument	
*k*	– coverage factor used in statistical analysis	
*L*	– thickness effect parameter	
µ-RS	– micro-Raman spectroscopy	
*m*	– measured mass	(kg)
NIMs	– nanoinsulation materials	
PCMs	– phase change materials	
PhF	– phenolic foam	
PIR	– polyisocyanurate	
PS	– polystyrene	
PUR	– polyurethane	
*q*	– density of heat flux through an insulation panel	(W·m^−2^)
*R*_0_	– extrapolated thermal resistance corresponding to panel thickness *d* = 0, in particular referring to product A, B, or C, respectively (after correction)	(m^2^·K·W^−1^)
*R*_0A_, *R*_0B_, *R*_0C_		
*R*′_0_	– extrapolated thermal resistance corresponding to panel thickness *d* = 0, in particular referring to product A, B, or C, respectively (before correction)	(m^2^·K·W^−1^)
*R*′_0A_, *R*′_0B_, *R*′_0C_		
*R*	– corrected thermal resistance (obtained by converging the corrected *λ* for a given panel thickness *d*)	(m^2^·K·W^−1^)
*R*_A_, *R*_B_, *R*_C_	– corrected thermal resistances referring to product A, B, or C, respectively	
*R*′	– apparent thermal resistance (as measured for a given panel thickness *d*)	(m^2^·K·W^−1^)
*R*′_A_, *R*′_B_, *R*′_C_	– apparent thermal resistance referring to product A, B, or C, respectively	
*R*_D_	– declared thermal resistance at *T*_m_ = 10 °C (assigned by a manufacturer to each panel of thickness *d*)	(m^2^·K·W^−1^)
*R*_DA_, *R*_DB_, *R*_DC_	– declared thermal resistance referring to product A, B, or C, respectively	
SIMs	– super insulating materials	
SRM	– standard reference material	
TDS	– technical data sheet provided by the manufacturer	
*T*_cm_	– crystalline melting temperature	(°C)
*T*_g_	– glass transition temperature	(°C)
*T*_m_	– average measurement (test) temperature	(°C)
*U*-value	– thermal transmittance (overall heat transfer coefficient)	(W·m^−2^·K^−1^)
VIMs	– vacuum insulation materials	
*x*, *y*	– panel dimensions: length, width	(m)
Δ*T*	– temperature difference between the “hot” and “cold” plates	(°C)
λ	– corrected thermal conductivity coefficient (resulted after correction of *λ*′ for a given panel of thickness *d*)	(W·m^−1^·K^−1^)
*λ*_A_, *λ*_B_, *λ*_C_	– corrected thermal conductivity coefficient referring to product A, B, or C, respectively	
*λ*′	– apparent thermal conductivity coefficient (as measured without Al-foil for a given panel thickness *d*)	(W·m^−1^·K^−1^)
*λ*′_A_, *λ*′_B_, *λ*′_C_, *λ*′_dotted_, *λ*′_grey_	– apparent thermal conductivity coefficient for a given product (as measured without Al-foil)	
*λ*″	– apparent thermal conductivity coefficient (as simulated or measured with Al-foil for a given panel thickness *d*)	
*λ*″_A_, *λ*″_B_, *λ*″_C_, *λ*″_dotted_, *λ*″_grey_	– apparent thermal conductivity coefficient for a given product (as simulated or measured with Al-foil)	
*λ*_D_	– thermal conductivity coefficient at *T*_m_ = 10 °C, as declared by a manufacturer (independent on thickness *d*)	(W·m^−1^·K^−1^)
*λ*_DA_, *λ*_DB_, *λ*_DC_	– declared thermal conductivity coefficient referring to product A, B, or C, respectively	
*λ* ^C^_SRM_	– SRM thermal conductivity coefficient (given in the Certificate for *T*_m_ = 10 °C)	(W·m^−1^·K^−1^)
*λ* ^M^_SRM_	– SRM thermal conductivity coefficient (measured with HFM FOX 600 at *T*_m_ = 10 °C)	(W·m^−1^·K^−1^)
*λ*_t_	– thermal transmissivity, horizontal asymptote of *λ*(*d*), reciprocal gradient of the *R*(*d*) linear fit (after correction)	(W·m^−1^·K^−1^)
*λ*_tA_, *λ*_tB_, *λ*_tC_	– thermal transmissivity, referring to product A, B, or C, respectively	
*λ*′_t_	– thermal transmissivity, horizontal asymptote of *λ*′(*d*), reciprocal gradient of the *R*′(*d*) oblique asymptote (before correction)	(W·m^−1^·K^−1^)
*λ*′_tA_, *λ*′_tB_, *λ*′_tC_	– thermal transmissivity referring to product A, B, or C, respectively	
*ρ*	– bulk density of polymeric insulation material (very low or low)	(kg·m^−3^)
*ρ*_A_, *ρ*_B_*, ρ*_C_	– bulk density value of product A, B, or C, respectively	
*ℑ*	– heat transfer factor, the effective conductivity coefficient (Supplementary Material part 1)	(W·m^−1^·K^−1^)
*ℑ*_A_, *ℑ*_B_, *ℑ*_C_	– heat transfer factor referring to product A, B, or C, respectively	
*Uncertainty*		
Δ	– absolute error, accuracy or absolute change of a quantity	
U(*𝒞*)	– expanded uncertainty of the correction parameter in the calculation of *𝒞*	(W·m^−1^·K^−1^)
*U*(*d*)	– expanded uncertainty of the sample thickness measurement in HFM FOX 600	(m)
*U*(*L*)	– expanded uncertainty of the thickness effect parameter calculation	
*U*(*m*)	– expanded uncertainty of the mass measurement	(kg)
*U*(*q*)	– expanded uncertainty of the heat flux density measurement	(W·m^−2^)
*U*(*x*) or *U*(*y*)	– expanded uncertainty of the *x*, *y* dimensions measurement	(m)
*U*(Δ*T*)	– expanded uncertainty of the temperature difference measurement	(°C)
*U*(*λ*)	– expanded uncertainty in determination of the thermal conductivity coefficient *λ*	(W·m^−1^·K^−1^)
*U*(*λ*′)	– expanded uncertainty in measurement of the thermal conductivity coefficient *λ*′	(W·m^−1^·K^−1^)
*U*(*λ*)/*λ*	– relative expanded uncertainty in determination of the thermal conductivity coefficient *λ*	(%)
*U*(*λ*′)/*λ*′	– relative expanded uncertainty in measurement of the thermal conductivity coefficient *λ*′	(%)
*U*(*λ*^C^_SRM_)	– expanded uncertainty in determination of the SRM thermal conductivity coefficient *λ*^C^_SRM_ (given in the Certificate for *T*_m_ = 10 °C)	(W·m^−1^·K^−1^)
*U*(*λ*^M^_SRM_)	– expanded uncertainty in measurement of the SRM thermal conductivity coefficient *λ*^M^_SRM_ (measured with HFM FOX 600 at *T*_m_ = 10 °C)	(W·m^−1^·K^−1^)
*U*(*ρ*)	– expanded uncertainty of the bulk density measurement	(kg·m^−3^)
*U*(*ρ*)/*ρ*	– relative expanded uncertainty of the bulk density measurement	(%)
σ	– standard deviation	
