# Supplementary material for: Change in Conductive–Radiative Heat Transfer Mechanism Forced by Graphite Microfiller in Expanded Polystyrene Thermal Insulation—Experimental and Simulated Investigations
Source: Materials (Basel). 2020 Jun 9;13(11):2626. doi: 10.3390/ma13112626 (PMC7321605; doi:10.3390/ma13112626)
Supplement: Supplementary file 1 [file materials-13-02626-s001.pdf]

materials MDPI

Supplementary Material

Change in Conductive–Radiative Heat Transfer Mechanism Forced by Graphite Microfiller in Expanded Polystyrene Thermal Insulation—Experimental and Simulated Investigations

Aurelia Blazejczyk [1,*], Cezariusz Jastrzebski [2] and Michał Wierzbicki [2]

1. Determination of the Thermo-Physical Parameters and the Thickness Limit

Measuring Thermal Parameters

After setting up the HFM FOX 600 (see Section 2.4.2), the one-dimensional heat flux density q was measured as a function of thickness d in all ranges (see Section 2.1), for each EPS A, B, and C panel. Next, based on this result, both the apparent thermal resistance $R'(d)$ and conductivity coefficient $\lambda'(d)$ were calculated by the HFM software, using formulae (1.1) and (1.2), respectively [28,29,32,33,35,36]:

$$R'(d) = \frac{\triangle T}{q(d)} \tag{1.1}$$

$$\lambda'(d) = \frac{q(d)}{\triangle T} d \tag{1.2}$$

In order to estimate relevance of the thickness effect and to carry out the correction procedure for the apparent thermal data (R' and λ'), only a few standard values of thickness d were initially selected. According to the Standard [35], the first value should be equal to 1/3 d_{max}, the second should be the average of the two outermost thickness values, and the last one should be equal to d_{max}. In the case of the EPS A product, due to greater density scattering (Figure 3), the standard thickness range was extended to form the sequence: 0.05, 0.06, 0.08, 0.10, 0.12, 0.14 (in m). The thickness values for the EPS B and EPS C were set as recommended: 0.04, 0.08, 0.12 and 0.05, 0.09, 0.13 (in m), respectively. To account for the $R'(d)$ data, initial fitting was carried out (within the domain of the selected thickness values) by applying the linear model:

$$R'(d) = \frac{1}{\lambda'_{\mathrm{t}}} d + R'_0 \tag{1.3}$$

For each type of EPS product, both the gradient $(\lambda'_{\mathrm{t}})^{-1}$ of the linear function (1.3) and the intercept R'_0 were obtained [33, 35]. The λ'_{t} parameter has a physical sense of thermal transmissivity. It corresponds, experimentally, to the upper bound of the λ' data set and, theoretically, to the horizontal asymptote of the function λ'(d). For the tested EPS A, B, and C products, the thermal transmissivity results were: $\lambda'_{\mathrm{tA}} = 0.0400$, $\lambda'_{\mathrm{tB}} = 0.0386$, and $\lambda'_{\mathrm{tC}} = 0.0314$, respectively (in W m^{-1} K^{-1}). The intercept R'_0 values were: $R'_{0\mathrm{A}} = 0.022$, $R'_{0\mathrm{B}} = 0.0316$, and $R'_{0\mathrm{C}} = 0.0043$, respectively (in m^2 K W^{-1}). Next, the heat transfer factor $\mathfrak{J}$ could be calculated in the frame of whole thickness range (from 0.02 to 0.18 m) by using the formula:

$$\mathfrak{J}(d) = \lambda'_{\mathrm{t}} \frac{1}{1 + \frac{R'_0 \lambda'_{\mathrm{t}}}{d}} \tag{1.4}$$

The factor $\mathfrak{I}$ has the same units as λ' (i.e., W $m^{-1} K^{-1}$) and is often called the effective or equivalent conductivity coefficient [33,35]; it has a physical sense of the heat flux density per temperature gradient normal to surface of the insulation panel, which separates the 'hot' and 'cold' surrounding environments. It depends on rate of the exchanged thermal energy per unit area at a given temperature difference. The factor $\mathfrak{I}$ also corresponds to the reciprocal gradient of a tangent line to the $R'(d)$ curve at each d.

Estimating the Thickness Limit d_L

Next, the function $\mathfrak{I}(d)$ for each EPS A, B, and C product could be used to determine the thickness effect function 1-L versus d, by the formula:

$$1 - L(d) = 1 - \frac{\mathfrak{I}(d)}{\lambda'_{\mathrm{t}}} = \frac{1}{1 + \frac{d}{R'_0 \lambda'_{\mathrm{t}}}} \tag{1.5}$$

which is a measure of the non-linearity of $\lambda'(d)$ or $R'(d)$ and allows us to estimate the relevance of the thickness effect. The parameter $1 - L$ can be represented as a value of the function (1.5) at the point d_L (thickness limit):

$$1 - L(d_{\mathrm{L}}) = \frac{1}{1 + \frac{d_{\mathrm{L}}}{R'_0 \lambda'_{\mathrm{t}}}} \tag{1.6}$$

which is the experimental tolerance for the non-linearity of $\lambda'(d)$ or $R'(d)$, with optimal value of 0.01 or 0.02, as recommended by the Standard [35].

After thermal measurement, including the full thickness range (Figure 4a), by applying the experimental condition ($1 - L = 0.02$), one can obtain the thickness limit, d_L, individually for each type of EPS product through the following procedure:

- iteratively increasing by the step by 0.001 m for the value of thickness d_i (i=1, 2, 3, ...), from the thinnest up;
- calculating the corresponding value of $1 - L_i(d_i)$ using formula (1.5); and
- checking by interpolation for whether the standard condition ($1 - L = 0.02$) is satisfied at a given d_i (if so, then d_L is assigned to d_i).

The accuracy of the d_L and L determination was estimated during the iteration procedure, by observing numerical variations of the second or third decimal place.

Correction Procedure

To obtain the proper values $\lambda(d)$ for the EPS A, B, and C panels, the measured values $\lambda'(d)$ were partially corrected in the range below the thickness limit d_L (where the thickness effect is relevant) [32], using the formula:

$$\lambda(d) = \frac{\lambda'(d)}{L(d)} = \lambda' \left(1 + \frac{R'_0 \lambda'_{\mathrm{t}}}{d}\right) \tag{1.7}$$

Next, the entire set of the $\lambda(d)$ values could be obtained by merging the corrected data (up to d_L) with the original measurements (above d_L). The function $\lambda(d)$ obtained in this procedure is shown in Figure 4b. Then, from the corrected $\lambda(d)$ values, we can calculate the average, $\langle\lambda\rangle$, representing the thermal conductivity of the given EPS product (also reported in Table 3).

The apparent thermal resistance $R'(d)$ correction was carried out indirectly, by converging the corrected $\lambda(d)$ into the $R(d)$ function, according to the relation:

$$R(d) = \frac{d}{\lambda(d)} \tag{1.8}$$

The thermal resistance $R(d)$ obtained this way is shown in Figure 5a.

In order to verify the correction procedure, the final fits were carried out within the whole domain of thickness by applying, in analogy to (1.3), the linear model:

$$R(d) = \frac{1}{\lambda_t} d + R_0 \tag{1.9}$$

For EPS B and C, the final fitting parameters were: $\lambda_{tB} = 0.0386$ and $\lambda_{tC} = 0.0314$ (in W m^{-1} K^{-1}), and $R_{0B} = 0.0316$ and $R_{0C} = 0.0043$ (in m^2 K W^{-1}), respectively. A good agreement could be observed between the initial and final fitting parameters, so the experimental correction results were considered accurate.

The corrected thermal resistance $R(d)$ data shown in Figure 5a includes the fitted lines and their equations, in gradient-intercept form.

2. Calculation of the Expanded Uncertainties

The uncertainty in measurements of physical quantities under study was evaluated according to the Guide of Uncertainty in Measurement.

The Expanded Uncertainty of the Bulk Density Measurement

This section extends Section 2.3 and Table 2, regarding the expanded uncertainty of the bulk density measurement.

The relative expanded uncertainty of the bulk density measurement $U(\rho)/\rho$ could be calculated, for each panel thickness *d*, according to the formula:

$$\left(\frac{U(\rho)}{\rho}\right)^2 = \left(\frac{U(m)}{m}\right)^2 + \left(\frac{U(x)}{x}\right)^2 + \left(\frac{U(y)}{y}\right)^2 + \left(\frac{U(d)}{d}\right)^2 \tag{2.1}$$

The expanded uncertainty of the bulk density measurement $U(\rho)$ could, then, be derived and calculated for each panel thickness, by rearranging (2.1) into:

$$U(\rho) = \rho \sqrt{\left(\frac{U(m)}{m}\right)^2 + \left(\frac{U(x)}{x}\right)^2 + \left(\frac{U(y)}{y}\right)^2 + \left(\frac{U(d)}{d}\right)^2} \tag{2.2}$$

The $U(\rho)$ values are shown in Figure 3, as error bars at each experimental point. For all tested EPS A, B, and C panels, the $U(\rho)$ data set results ranged from 5.8×10^{-1} kg m^{-3} (at $d = 0.02$ m) to 6.0×10^{-2} kg m^{-3} (at $d = 0.18$ m). The uncertainty values decreased with an increase in thickness.

The Expanded Uncertainties of the Thermal Conductivity Measurement

This section extends Section 2.4.3 and Table 3, regarding the expanded uncertainty of the apparent thermal conductivity $U(\lambda')$ measurement and the expanded uncertainty of the corrected thermal conductivity $U(\lambda)$ determination.

The relative expanded uncertainty of the apparent thermal conductivity measurement $U(\lambda')/\lambda'$ could be calculated, for each panel thickness *d*, according to the formula:

$$\left(\frac{U(\lambda')}{\lambda'}\right)^2 = \left(\frac{U(q)}{q}\right)^2 + \left(\frac{U(d)}{d}\right)^2 + \left(\frac{U(\Delta T)}{\Delta T}\right)^2 + \left(\frac{U(\lambda^{C}{}_{SRM})}{\lambda^{C}{}_{SRM}}\right)^2 + \left(\frac{U(C)}{\lambda^{M}{}_{SRM}}\right)^2 \tag{2.3}$$

The relative expanded uncertainty of the corrected thermal conductivity determination $U(\lambda)/\lambda$ could be calculated, for each panel thickness *d*, according to the formula:

$$\left(\frac{U(\lambda)}{\lambda}\right)^2 = \left(\frac{U(q)}{q}\right)^2 + \left(\frac{U(d)}{d}\right)^2 + \left(\frac{U(\Delta T)}{\Delta T}\right)^2 + \left(\frac{U(\lambda^{C}{}_{SRM})}{\lambda^{C}{}_{SRM}}\right)^2 + \left(\frac{U(C)}{\lambda^{M}{}_{SRM}}\right)^2 + \left(\frac{U(L)}{L}\right)^2 \tag{2.4}$$

The expanded uncertainties of the apparent and corrected thermal conductivities $U(\lambda')$ and $U(\lambda)$ could then be derived and calculated by rearranging (2.3) and (2.4) for each panel thickness. The expanded uncertainties $U(\lambda')$ and $U(\lambda)$ are shown in Figures 3a and 3b, respectively, as error bars at each experimental point. For all tested EPS A, B, and C panels, the $U(\lambda')$ data set ranged from 5.3×10^{-4} (at d = 0.02 m) to 1.4×10^{-3} W m^{-1} K^{-1} (at d = 0.18 m); for EPS A and B, $U(\lambda)$ ranged from 8.1×10^{-4} (at d = 0.05 m) to 1.4×10^{-3} W m^{-1} K^{-1} (at d = 0.18 m). In general, the uncertainty values increased with thickness; yet, in the corrected (EPS A and B only) region $d < d_L$, the opposite trend was observed.

3. Method for Separating Components of Total Thermal Conductivity

Procedure with Reasoning (Radiation Contribution)

To find the radiation contribution for each EPS product and each panel thickness (from 0.01 to 0.10 m), the thermal conductivity values (measured with and without Al-foil) were implemented, from [42], by estimating the co-ordinates of the experimental points.

The change in apparent conductivity after applying the Al-foil to each EPS panel was, then, calculated as:

$$\Delta\lambda'_{\text{dotted}} = \lambda''_{\text{dotted}} - \lambda'_{\text{dotted}} \quad (3.1)$$

$$\Delta\lambda'_{\text{grey}} = \lambda''_{\text{grey}} - \lambda'_{\text{grey}} \quad (3.2)$$

The above quantities, ignoring the negative sign, were assumed as basically corresponding to the radiation components of total thermal conductivity for the 'dotted' and 'grey' EPS, as the measurements using Al-foil allowed us to effectively block thermal radiation by reflection (primary, emitted by the HFM 'hot' plate; and secondary, radiation generated across the insulation). It might be reduced by more than 96%, assuming that emissivity (either of the polished or rough Al-foil side) is less than 0.04 (see Section 2.4.2). Thus, the Al-foil practically removes the radiation from the heat flux, while does not affect thermal conduction through the EPS insulation.

The tested EPS B thermal conductivity, as a simulated result of a measurement with Al-foil, could be then calculated by:

$$\lambda''_{\text{B}} = \lambda'_{\text{B}} \cdot \left(1 - \frac{|\Delta\lambda'_{\text{dotted}}|}{\lambda'_{\text{dotted}}}\right) \quad (3.3)$$

Additionally, since the measured 'grey' EPS conductivity was almost the same with or without Al-foil [42], as well as the density and GMP concentration of EPS C being the same, the tested EPS C thermal conductivity (as a simulated result of a measurement with Al-foil) could be obtained from:

$$\lambda''_{\text{C}} = \lambda'_{\text{C}} \quad (3.4)$$

Based on (3.3) and (3.4), the change in apparent conductivity expected after applying the Al-foil on each tested EPS panel could be calculated by:

$$\Delta\lambda'_{\text{B}} = \lambda''_{\text{B}} - \lambda'_{\text{B}} \quad (3.5)$$

$$\Delta\lambda'_{\text{C}} = \lambda''_{\text{C}} - \lambda'_{\text{C}} \quad (3.6)$$

By analogy, the above quantities were assumed as basically corresponding to the radiation components of the total thermal conductivity for EPS B and C. Then the following values of the percentage decrease in thermal conductivity caused by Al-foil:

$$\frac{|\Delta\lambda'_{\text{dotted}}|}{\lambda'_{\text{dotted}}} \cdot 100\% \ \ \text{(for 'dotted' EPS)} \quad (3.7)$$

$$\frac{|\Delta\lambda'_{\mathrm{B}}|}{\lambda'_{\mathrm{B}}} \cdot 100\% \ \ \text{(expected for EPS B)} \tag{3.8}$$

$$\frac{|\Delta\lambda'_{\mathrm{grey}}|}{\lambda'_{\mathrm{grey}}} \cdot 100\% \approx \frac{|\Delta\lambda'_{\mathrm{C}}|}{\lambda'_{\mathrm{C}}} \cdot 100\% \approx 0 \ \ \text{(for ‘grey’ EPS and expected for EPS C)} \tag{3.9}$$

were assumed as practically corresponding to the thermal radiation (percentage) contributions for each EPS product, respectively.

Procedure with Reasoning (Conduction Contributions)

Subsequently, the percentage contribution of the solid matrix thermal conduction in the total heat transfer could be calculated by the following expressions, for each EPS product:

$$[1 - \left(\frac{|\Delta\lambda'_{\mathrm{dotted}}|}{\lambda'_{\mathrm{dotted}}} + \frac{\lambda'_{\mathrm{air}}}{\lambda'_{\mathrm{dotted}}}\right)] \cdot 100\% \tag{3.10}$$

$$[1 - \left(\frac{|\Delta\lambda'_{\mathrm{grey}}|}{\lambda'_{\mathrm{grey}}} + \frac{\lambda'_{\mathrm{air}}}{\lambda'_{\mathrm{grey}}}\right)] \cdot 100\% \tag{3.11}$$

$$[1 - \left(\frac{|\Delta\lambda'_{\mathrm{B}}|}{\lambda'_{\mathrm{B}}} + \frac{\lambda'_{\mathrm{air}}}{\lambda'_{\mathrm{B}}}\right)] \cdot 100\% \tag{3.12}$$

$$[1 - \left(\frac{|\Delta\lambda'_{\mathrm{C}}|}{\lambda'_{\mathrm{C}}} + \frac{\lambda'_{\mathrm{air}}}{\lambda'_{\mathrm{C}}}\right)] \cdot 100\% \tag{3.13}$$

where the measured conduction component of air was $\lambda'_{air} \approx \lambda_{air} \approx 0.025$ W m^{-1} K^{-1}, the value given for still air at 10 °C and at atmospheric pressure [30]. The following expressions were obtained for each EPS product:

$$\frac{\lambda'_{\mathrm{air}}}{\lambda'} \cdot 100\% \ \ (\text{for } \lambda' = \lambda'_{\mathrm{dotted}}\,;\ \lambda'_{\mathrm{grey}}\,;\ \lambda'_{\mathrm{B}}\,; \text{and } \lambda'_{\mathrm{C}}) \tag{3.14}$$

providing the percentage contribution of air thermal conduction in total heat transfer.

© 2020 by the authors. Submitted for possible open access publication under the terms and conditions of the Creative Commons Attribution (CC BY) license (http://creativecommons.org/licenses/by/4.0/).
